# Mouse Plasminogen Has Oxidized Phosphatidylcholine Adducts That Are Not Metabolized by Lipoprotein-Associated Phospholipase A_2_ under Basal Conditions

**DOI:** 10.3390/ijms11125339

**Published:** 2010-12-22

**Authors:** Celina Edelstein, Ditta Pfaffinger, Ethan C. Reichert, Diana M. Stafforini, Angelo M. Scanu

**Affiliations:** 1 Department of Medicine, University of Chicago, Chicago, IL 60637, USA; E-Mails: celina@medicine.bsd.uchicago.edu (C.E.); dpfaffin@medicine.bsd.uchicago.edu (D.P.); 2 Huntsman Cancer Institute University of Utah, Salt Lake City, UT 84112, USA; E-Mails: ethan.reichert@hci.utah.edu (E.C.R.); diana.stafforini@hci.utah.edu (D.M.S.); 3 Department of Internal Medicine, University of Utah, Salt Lake City, UT 84132, USA; 4 Department of Biochemistry and Molecular Biology, University of Chicago, Chicago, IL 60637, USA

**Keywords:** mouse plasminogen, oxidized phosphatidylcholine, lipoprotein-associated phospholipase A_2_, oxidized phopholipids

## Abstract

We previously showed that plasminogen (Plg) isolated from the plasma of normal human subjects contains 1–2 moles of oxidized phosphatidylcholine (oxPtdPC) adducts/mole of protein. Moreover, we suggested that these species are generated at the hepatic site and speculated that they may play a role in the reported cardiovascular pathogenicity of Plg. We aimed to determine whether mouse Plg also harbors linked oxPtdPCs and whether these molecules are metabolized by lipoprotein-associated phospholipase A_2_/PAF acetylhydrolase (Lp-PLA_2_/PAF-AH), an enzyme specific for hydrolysis of oxPtdPCs. We determined the total concentration of Plg in plasma samples from control (WT) and Lp-PLA_2_-deficient (KO) mice, we isolated Plg, and assessed its content of oxPtdPCs by immunoblot analyses. We also evaluated whether human recombinant Lp-PLA_2_ metabolized Plg-linked oxPtdPCs *in vivo* and *in vitro*. WT and KO mice expressed comparable levels (14.4–15.8 mg/dL) of plasma Plg, as determined by ELISA. We observed no differences in the content of oxPtdPC in Plg isolated from the two mouse strains and in parallel no changes in oxPtdPC content in mouse Plg following incubation with pure recombinant Lp-PLA_2_. Plg from mouse plasma contains oxPtdPC adducts that are not affected by the action of Lp-PLA_2_, suggesting that linkage to Plg protects oxPtdPCs from metabolism during their transport in the plasma. This modification may have important physio-pathological implications related to the function of Plg, oxPtdPCs, or both.

## 1. Introduction

We previously showed that oxidized phosphatidylcholine (oxPtdPC) is chemically linked to naturally occurring human apolipoprotein (a) [apo(a)] and that this linkage involves 1 to 2 lysines in kringle V located in the *C*-terminal domain of this apolipoprotein [1]. We subsequently showed that oxPtdPCs are linked to apo(a) in a 2:1 ratio that is not affected by plasma Lp(a) levels or apo(a) size polymorphisms [2]. We also reported that in healthy subjects, linked oxPtdPCs do not originate from plasma LDL or the LDL component of Lp(a) [2]. Recently, we reported that naturally occurring human Plg, a multi-kringle structure that shares structural and genetic features with apo(a), contains about 2 mol of oxPtdPCs/mol [3]. We also showed that human HepG2 cells secrete Plg containing linked oxPtdPCs, [3] suggesting that these modified lipids are covalently linked to Plg in the liver [2].

The purpose of the present study was to determine whether mouse Plg, which is 79% homologous to human Plg, contains linked oxPtdPCs and whether these oxidized lipid species are subject to metabolism by lipoprotein-associated phospholipase A_2_ (Lp-PLA_2_) [4]. This enzyme, also known as platelet-activating factor (PAF) acetylhydrolase (PAF-AH) has specificity for hydrolysis of *sn*-2 acyl groups present in biologically active phospholipids such as PAF and oxPtdPCs, and has been proposed to have both pro- and anti-inflammatory functions [5]. Previous studies were focused on the ability of Lp-PLA_2_ to hydrolyze free and non-covalently-bound, lipoprotein-associated oxPtdPCs in human [6] and mouse [7,8] plasma. Thus, the issue of whether Lp-PLA_2_ metabolizes oxPtdPC-protein adducts, particularly in *in vivo* settings, is a novel area of investigation.

## 2. Results

### 2.1. Plasminogen from WT and KO Mice

**Figure 1.**
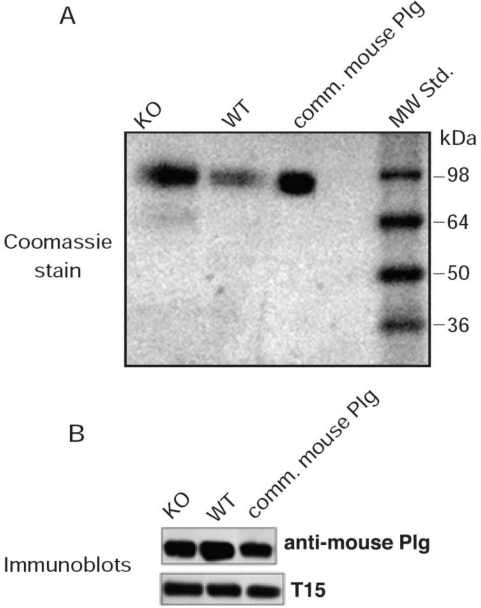
Purification of mouse Plg. (**A**) Coomassie-stained gel depicting the behavior of purified mouse Plg from WT and KO mice on a non-reduced 4–12% SDS-PAGE gel. Lane KO, 5 μg of protein applied; lane WT, 2 μg of protein applied; comm. mouse Plg, 5 μg of protein applied; (**B**) Immunoblot of purified Plg from a commercial source and from WT and KO mice, probed with anti-mouse Plg antibodies and T15.

The concentration of Plg in the plasma of WT and KO mice as determined by ELISA was 15.8 ± 1.2 and 14.4 ± 1.3 mg/dL, respectively (mean ± SD of four determinations in duplicate from four mice). From each plasma sample, Plg was isolated by lysine-Sepharose affinity chromatography. In both cases, the product contained a single band migrating in the same position as standard Plg when analyzed on 4–12% SDS-PAGE gradient gels stained with Coomassie Blue (Figure 1A). In addition, Plg samples from WT and KO mice reacted against anti-mouse Plg (Figure 1B).

### 2.2. Mouse Plasminogen Contains Covalently Linked OxPtdPCs

To evaluate whether mouse Plg contained OxPtdPCs we subjected Plg isolated from WT and KO mice to immunoblot analyses using T15. We found that both sets of samples reacted strongly with the OxPtdPC-specific monoclonal antibody (Figure 1B). Pre-treatment with denaturing agents, extraction with organic solvents and 1–2 cycles of freezing/thawing failed to affect T15 reactivity (not shown). These results indicated that OxPtdPCs are covalently linked to Plg.

### 2.3. Lp-PLA_2_ Does Not Cleave OxPtdPC Linked to Plasminogen

**Figure 2.**
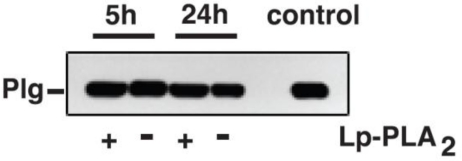
Lp-PLA_2_ does not metabolize OxPtdPCs linked to Plg. A, T15 immunoblot of mouse Plg from WT and KO mice, before and after 5 h and 24 h incubations with active Lp-PLA_2_.

To investigate whether Plg-bound oxPtdPCs are metabolized by Lp-PLA_2_ *in vitro* we incubated Plg isolated from WT and KO mouse plasma with a pure, enzymatically active, preparation of human Lp-PLA_2_. Both sets of samples exhibited the same T15 reactivity as incubated (24 h at 37 °C), but untreated, controls (Figure 2). These *in vitro* studies strongly suggested that Lp-PLA_2_ does not metabolize oxPtPC linked to Plg.

**Figure 3.**
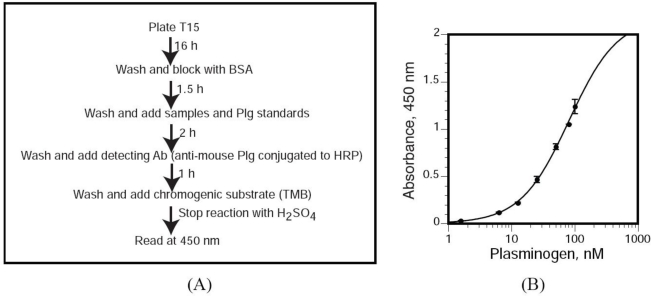
Sandwich ELISA for detection of Plg-bound OxPtdPC. (**A**) We developed a sandwich ELISA for the detection of OxPtdPC linked to Plg in which T15 was the capture antibody and anti-mouse Plg conjugated to HRP was the detection antibody. (**B**) Dose response curve obtained with purified mouse Plg. Vertical bars through the data points represent means ± SD.

To further investigate this issue, we quantified the oxPtdPC molecules bound to Plg isolated from WT and KO mice by ELISA (see “Methods”) (Figure 3A). The assay was highly reproducible over a wide range of concentrations (1.56 to 100 nmol/L, Figure 3B). From the values obtained we calculated an oxPtdPC:Plg ratio of one for both Plg samples, establishing that under basal conditions, deletion of Lp-PLA_2_ does not affect the extent of Plg conjugation with oxPtdPCs.

## 3. Experimental Section

### 3.1. Materials and Methods

The studies in mice were carried out in strict accordance with the recommendations in the Guide for the Care and Use of Laboratory Animals of the National Institutes of Health. The protocol was approved by the committee on the Ethics of Animal Experiments of the University of UTAH (animal welfare assurance number A3031-01).

### 3.2. Materials

BSA, Tween-20, SDS, ε-amino caproic acid, 4-(2-Aminoethyl)-benzene sulfonylfluoride, and *N*-α-tosyl-L-lysine chloromethylketone hydrochloride were from Sigma-Aldrich Chemical Co. (St. Louis, MO, USA). Immobilon-P membranes were from Millipore Corp. (Billerica, MA, USA) and Superblock blocking buffer and the Supersignal^®^ West Dura Extended Duration Substrate were purchased from Thermo Scientific (Rockford, IL). Coomassie staining solution (Page Blue) was from Fermentas Inc. (Glen Burnie, MD, USA). Precast acrylamide gels were from Invitrogen Corp. (Carlsbad, CA, USA). All chemicals were of reagent grade.

Human recombinant Lp-PLA_2_ (Pafase^®^) was a generous gift from ICOS Corporation (Bothell, WA, USA).

### 3.3. Antibodies

The murine T15 antibody-secreting cell line, BH8 (IgM) that reacts with oxPtdPC antigens was a gift from Dr. John F. Kearney (University of Alabama) and is heretofore referred to as T15. This cell line was maintained in the Frank W. Fitch Antibody Facility of the University of Chicago. The antibodies in the culture medium were isotyped and contained a high titer of IgM antibodies (greater than 90%) as verified by the major Coomassie stained band on SDS-PAGE.

Affinity purified rabbit anti-mouse Plg polyclonal antibodies were obtained from Cell Sciences (Canton, MA, USA). Horseradish peroxidase (HRP)-labeled secondary antibodies [goat anti-mouse IgM (μ chain specific) and donkey anti-rabbit IgG] were from Sigma-Aldrich.

### 3.4. Plasminogen Isolation and Quantitation

Plasma from healthy mice was utilized to purify Plg using the procedure of Deutsch and Mertz [9], followed by purification on G25 Sepharose columns. We also employed commercial preparations of mouse Plg purchased from Enzyme Research Laboratories (South Bend, IN, USA). Quantitative determination of Plg in mouse plasma and in purified samples was conducted using an ELISA kit (Kamiya Biomedical Co., Seattle, WA, USA). The Plg standard concentrations ranged from 6.25 to 200 ng/mL.

### 3.5. Delipidation of Plasminogen

Lipids were extracted from purified Plg by a modified Bligh and Dyer method [10] in that chloroform was replaced by anhydrous diethyl ether in a ratio of 1:2 v/v (ether:methanol). Plg in 0.8 mL 50 mM Tris-HCl containing 0.1 M NaCl, pH 7.4 was added to the ethyl ether-methanol mixturedropwise and rotated at 4 °C for 6 h. The mixture was then centrifuged and the precipitated protein washed three times with diethyl ether. The washed precipitate was dried with argon gas and dissolved in the Tris buffer. The protein recovery was close to 85% of the original starting material. An alternative delipidation technique was also carried out using a 3:2 v/v mixture of ethanol:diethyl ether as already published for apo(a) [1]. In both delipidation techniques the Plg was soluble in aqueous buffers.

### 3.6. Electrophoretic Methods

SDS-PAGE (gradients of 4–12% polyacrylamide) was performed on a Novex system (Novex, San Diego, CA) for 1.5 h at constant voltage (120 V) at 22 °C, as described [1,2]. The samples were prepared by heating at 95 °C for 5 min in sample buffer [94 mM phosphate buffer (pH 7.0), 1% SDS and 2 M urea, with or without 3% mercaptoethanol]. Following electrophoresis, the gels were placed on Immobilon-P sheets (Millipore Corp., Bedford, MA) previously wetted in 48 mM Tris, 39 mM glycine (pH 8.9). Blotting was performed on a horizontal semi-dry electroblot apparatus (Amersham Biosciences) at 0.8–1 mA/cm^2^ for 45 min at 23 °C.

### 3.7. Immunoblot Analyses

After electroblotting, the Immobilon-P sheets were blocked in Superblock (Thermo Scientific, Inc. Rockford, IL. USA) for 1 h at 23 °C followed by incubation with rabbit anti-mouse Plg IgG or, in the case of T15, by incubation in 10% Superblock for 18 h at 4 °C. Bound antibodies were visualized using secondary antibodies conjugated to HRP; donkey anti-rabbit IgG for Plg, and goat anti-mouse IgM (μ-chain specific) for oxPtdPC. The blots were developed with Supersignal^®^ West Dura Extended Duration Substrate from Thermo Scientific, Inc. (Rockford, IL).

### 3.8. T15 ELISA for Quantitation of OxPtdPC

We utilized a novel sandwich ELISA by which we measured T15 immunoreactivity in Plg isolated from mouse plasma. Polystyrene microtiter plates (flat-bottom 96-well EIA plates) were coated with 100 μL of T15 [400 ng/well, dissolved in TBS buffer [50 mmol/L Tris-HCl, 0.15 mol/L NaCl (pH 7.5)] and incubated overnight at room temperature, in 10 mmol/L Tris, 0.15 mol/L NaCl (pH 7.6). Unbound antibodies were removed by washing with TBS supplemented with 0.1% BSA and 0.02% Tween-20. Non-specific binding sites were blocked with 1% BSA in TBS for 1.5 h. After three washes with TBS supplemented with 0.02% Tween-20 (TBST), we added 100 μL of each dilution of the mouse Plg standard and samples (diluted in TBST) and incubated the plates for 2 h at room temperature. After three washes with TBST, bound Plg was detected by incubation with a specific, HRP-conjugated anti-mouse Plg polyclonal antibody for 1 h in TBST. The wells were washed three times with TBST, the chromogenic substrate 3,3′,5,5′-tetramethylbenzidine was added and, after an appropriate incubation period at room temperature, the reactions were stopped with 3 mol/L H_2_SO_4_. T15 immunoreactivity was quantitated by assessing the absorbance at 450 nm using a Versamax microplate reader (Molecular Devices, Sunnyvale, CA). The concentrations of the Plg standard ranged from 1.56 to 100 nmol/L.

### 3.9. Defining T15 Equivalents

Based on the standard curve, the absorbance of each sample reacting with T15 was converted to nmol/L of Plg. This number was divided by the concentration (nmol/L) of Plg in each mouse sample. This ratio was defined as the “T15 equivalent”.

### 3.10. Targeted Disruption of the Mouse Lp-PLA_2_ (*Pla2g7*) Gene

The generation of *Pla2g7^−/−^* mice took place at the University of Utah and was recently described in detail [11] Briefly, a targeting vector lacking exons 3 and 4 of the mouse *Pla2g7* gene was introduced into embryonic stem cells and a resulting chimeric male was bred to C57BL/6J females. The progeny was genotyped as previously described [11].

### 3.11. Incubation of Mouse Plg with Lp-PLA_2_

Mouse Plg (1 to 3 μg) was incubated with recombinant Lp-PLA_2_ (4 μg) in PBS for up to 24 h at 37 °C. The reactions were stopped with Pefabloc and the samples were stored frozen at −20 °C until subsequent analyses.

## 4. Discussion

A novel finding of our current studies was that freshly isolated mouse Plg and commercial sources contain chemically linked oxPtdPCs. The chemical linkage, likely of the Schiff base nature, was suggested by the observations that T15 immunoreactivity was present in fresh and frozen samples, was not altered by extensive delipidation with organic solvents or by boiling in the presence of SDS. Of note, our observations were made under basal, unstimulated conditions, and paralleled our previous results in both apo(a) and Plg isolated from normal human plasma [2,3]. Together, our findings challenge the belief that oxPtdPC generation only occurs in response to pro-oxidant/pro-inflammatory events which would presumably be quite limited in healthy subjects and in unstimulated mice [4]. A plausible explanation is that adduct formation occurs during early, pre-secretory stages of Plg generation. In previous studies we proposed a model whereby the syntheses of human apo(a) [1] and human Plg [3] may be accompanied, or shortly followed, by oxPtdPC linkage to newly synthesized proteins. We now speculate that the liver provides a minimally pro-oxidant/pro-inflammatory microenvironment exquisitely sensed by a lysine residue(s) in the Plg molecule, leading to the formation of oxPtdPC-Plg adducts.

A second novel observation from quantitative and metabolic standpoints was that the extent of Plg conjugation with oxPtdPC was not affected by Lp-PLA_2_. This suggests that under basal conditions, Lp-PLA_2_ does not play a critical role in the metabolism of Plg-bound oxPtdPCs during transport in the circulation. Lack of activity may be due to peculiar Plg conformations that prevent the candidate linkage sites from interacting with Lp-PLA_2_, an interpretation consonant with *in vitro* studies in which oxPtdPC was not cleaved when mouse Plg was incubated with Lp-PLA_2_. We recognize that in our *in vitro* studies we utilized a recombinant rather than a naturally occurring Lp-PLA_2_ that, in the mouse plasma, under normal conditions is expected to be essentially all bound to HDL [4]. We believe, however, that the validity of our *in vitro* experiments is supported by the results of our *in vivo* studies in KO mice. In our previous study, cleavage of oxPtdPCs linked to human Plg occurred but only under conditions of a high enzyme to substrate ratio and prolonged times of incubation [3].

Further studies are needed to determine whether the number of oxPtdPCs linked to Plg increases in settings of elevated oxidant and/or inflammatory stress and to assess whether Lp-PLA_2_ modulates this process. These analyses may provide important clues to explain why Plg has pathological cardiovascular properties, an issue emerging from recent studies in Plg-deficient mice [12]. In this regard, plasma from transgenic mice expressing human apo(a) contain Lp(a)/apo(a) with bound oxPtdPCs proposed to contribute to uremia-induced atherosclerosis [13,14]. However, the potential contribution by the oxPtdPC-Plg adducts cannot be ruled out. Considering our observation that Plg is the major oxPtdPC carrier in the circulation, contrary to an early belief [15], we may speculate that Plg modulates OxPtdPC metabolism in the circulation, in physiological and possibly pathological settings.

In the current and previous studies, the number of linked oxPtdPCs was limited to 1 or 2. In the case of apo(a), the modification involved specific microdomains within kringle V [1,3], suggesting that a kringle repeat(s) may also participate in linkage of OxPtdPCs to Plg. Under physiological conditions it is possible that the formation of Plg adducts reflects a mechanism to shield potential pro-inflammatory OxPtdPCs from metabolism during transport in the circulation. To rigorously test this, it would be ideal to compare the properties of Plg adducts harboring various amounts of oxPtdPCs. Unfortunately, given the chemical nature of the oxPtdPC-Plg linkage, the lipid component is not amenable to extraction by organic solvents; drastic procedures would likely result in denatured, unsuitable bio-products. Also unfortunate is the fact that Lp-PLA_2_ cannot be utilized to generate OxPtdPC-free Plg. Mutagenic approaches should prove useful, but it will be necessary to first identify the oxPtdPC linkage site on Plg.

## 5. Conclusions

In conclusion, under physiological conditions, mouse Plg, like its human counterpart, carries chemically linked oxPtdPCs that are not cleaved by Lp-PLA_2_, an enzyme known to be active on oxidized phospholipids associated with plasma lipoproteins. Future studies will elucidate the molecular basis for lack of recognition and/or metabolism of Plg-bound oxPtdPCs by Lp-PLA_2_. These studies should provide a platform to assess possible relationships between oxPtdPCs, Plg, and Lp-PLA_2_ under stressed and pathological conditions. Work along these lines may have broad physio-pathological implications, including issues related to the function of the pro-atherogenic lipoprotein Lp(a).

## References

[1] Edelstein C, Pfaffinger D, Hinman J, Miller E, Lipkind G, Tsimikas S, Bergmark C, Getz GS, Witztum JL, Scanu AN (2003). Lysine-phosphatidylcholine adducts in kringle V impart unique immunological and potential pro-inflammatory properties to human apolipoprotein(a). J. Biol. Chem.

[2] Edelstein C, Philips B, Pfaffinger D, Scanu AM (2009). The oxidized phospholipids linked to human apolipoprotein(a) do not derive from circulating low-density lipoproteins and are probably of cellular origin. FASEB J.

[3] Edelstein C, Pfaffinger D, Yang M, Hill JS, Scanu AM (2010). Naturally occurring human plasminogen, like genetically related apolipoprotein(a), contains oxidized phosphatidylcholine adducts. Biochim. Biophys. Acta.

[4] Stafforini DM (2009). Biology of platelet-activating factor acetylhydrolase (PAF-AH), lipoprotein associated phospholipase A_2_. Cardiovasc. Drugs Ther.

[5] Karasawa K (2006). Clinical aspects of plasma platelet-activating factor-acetylhydrolase. Biochim. Biophys. Acta.

[6] Davis B, Koster G, Douet LJ, Scigelova M, Woffendin G, Ward JM, Smith A, Humphries J, Burnand KG, Macphee CH, Postle AD (2008). Electrospray ionization mass spectrometry identifies substrates and products of lipoprotein-associated phospholipase A_2_ in oxidized human low density lipoprotein. J. Biol. Chem.

[7] Quarck R, de Geest B, Stengel D, Mertens A, Lox M, Theilmeier G, Michiels C, Raes M, Bult H, Collen D, van Veldhoven P, Ninio E, Holvoet P (2001). Adenovirus-mediated gene transfer of human platelet-activating factor-acetylhydrolase prevents injury induced neointima formation and reduces spontaneous atherosclerosis in apolipoprotein E-deficient mice. Circulation.

[8] Theilmeier G, de Geest B, van Veldhoven PP, Stengel D, Michiels C, Lox M, Landeloos M, Chapman MJ, Ninio E, Collen D (2000). HDL-associated PAF-AH reduces endothelial adhesiveness in apoE^−/−^ mice. FASEB J.

[9] Deutsch DG, Mertz ET (1970). Plasminogen: Purification from plasma by affinity chromatography. Science.

[10] Bligh EG, Dyer WJ (1959). A rapid method of total lipid extraction and purification. Can. J. Biochem. Physiol.

[11] Lu J, Pierce M, Franklin A, Jilling T, Stafforini DM, Caplan M (2010). Dual role of endogenous platelet-activating factor acetylhydrolase in a murine model of necrotizing enterocolitis. Pediatric Res.

[12] Plow EF, Hoover-Plow J (2004). The functions of plasminogen in cardiovascular disease. Trends Cardiovasc. Med.

[13] Pederson TX, McCormick SP, Tsimikas S, Bro S, Nielsen LB (2010). Lipoprotein(a) accelerates atherosclerosis in uremic mice. J. Lipid Res.

[14] Schneider M, Witztum JL, Young SG, Ludwig EH, Miller ER, Tsimikas S, Curtiss LK, Marcovina SM, Taylor JM, Lawn RM (2005). High-level lipoprotein(a) expression in transgenic mice: evidence for oxidized phospholipids in lipoprotein(a) but not in low density lipoproteins. J. Lipid Res.

[15] Tsimikas S, Witztum JL (2008). The role of oxidized hospholipids in mediating lipoprotein(a) atherogenicity. Curr. Opin. Lipidol.

